# A new perspective on amino acid infusion: from perioperative parenteral nutrition to renal protection

**DOI:** 10.1186/s40981-024-00723-2

**Published:** 2024-06-13

**Authors:** Yoshitaka Aoki, Yoshiki Nakajima

**Affiliations:** Department of Anesthesiology and Intensive Care Medicine, Hamamatsu University School of Medicine, 1-20-1 Handayama, Chuo-Ku, Hamamatsu, Shizuoka 431-3192 Japan

**Keywords:** Amino Acids, Body Temperature, Parenteral Nutrition, Acute Kidney Injury

To the Editor

We have read with great interest the article by Kazawa et al. on the use of amino acid infusions to prevent acute kidney injury (AKI) following cardiac surgery, published in your esteemed journal [1]. The authors have demonstrated the therapeutic potential of amino acid infusion for preventing AKI after cardiac surgery through a meticulously designed randomized controlled trial, intriguingly broadening its clinical applications beyond traditional parenteral nutrition. We commend the authors for their earnest research and seek to further discuss perioperative amino acid infusion.

In the early 2000s, Mizobe and colleagues extensively researched amino acid infusions, publishing their findings in top-tier journals [2–4]. These studies primarily focused on the metabolic effects of amino acid infusions during the perioperative period, including energy production and their impact on patient body temperature, thereby laying the groundwork for further exploration of the clinical utility of amino acids. Building upon this historical context, we conducted a systematic review to evaluate the effects of amino acid infusions on patient outcomes across multiple studies [5]. Our analysis revealed that patients receiving amino acid infusions experienced a mean increase in body temperature of 0.46 °C compared to control groups. For other outcomes, amino acid infusions were associated with reduced frequency of shivering, shorter post-operative intubation times, and shorter length of hospital stay. However, as of our systematic review in 2015, there were no reported effects of amino acid infusions on the prevention of AKI.

An interesting aspect of the study by Kazawa et al. is the potential renal protective effects of amino acids. This research suggests that amino acid infusions may positively impact urine output and estimated glomerular filtration rate (eGFR) in the days following surgery, providing a protective mechanism against AKI, a significant risk associated with cardiac surgery. Moving from traditional perioperative care, which primarily focuses on maintaining physical parameters such as body temperature, to exploring biochemical interventions that could improve patient outcomes is both timely and necessary. Given that AKI remains a challenging complication of cardiac surgery, understanding and utilizing the renal protective potential of amino acids could lead to substantial improvements in patient care.

We recognize that amino acid dosage is crucial for therapeutic outcomes. Kazawa et al. used 60 g/day, the dose recommended in Japan, while international studies, such as Pu et al., often used higher doses, like 100 g/day [6]. This suggests that the relatively low amino acid dosage recommended by the Japanese Ministry of Health, Labour and Welfare may still have clinical benefits. Further major strengths of this study include the implementation of appropriate statistical analyses conducted by experts, the use of Research Electronic Data Capture software (REDCap®), and adherence to the Kidney Disease Improving Global Outcomes (KDIGO) guidelines, making it commendable for several key aspects.

We acknowledge the contributions of Kazawa et al. and recommend further research, including multicentric trials with diverse patient populations, to confirm these findings and determine optimal protocols for amino acid infusion in the perioperative setting.

## Data Availability

Not applicable.

## Abbreviations

AKI: Acute kidney injury
eGFR: Estimated glomerular filtration rate
KDIGO: Kidney Disease Improving Global Outcomes
